# Mitochondrial permeability transition pore induction is linked to formation of the complex of ATPase C-subunit, polyhydroxybutyrate and inorganic polyphosphate

**DOI:** 10.1038/cddiscovery.2016.70

**Published:** 2016-12-05

**Authors:** P A Elustondo, M Nichols, A Negoda, A Thirumaran, E Zakharian, G S Robertson, E V Pavlov

**Affiliations:** 1 Department of Physiology and Biophysics, Faculty of Medicine, Dalhousie University, Halifax, NS, B3H 4R2 Canada; 2 Departments of Psychiatry and Pharmacology, Brain Repair Centre, Faculty of Medicine Dalhousie University, Halifax, NS, B3H 4R2f Canada; 3 Department of Cancer Biology and Pharmacology, University of Illinois College of Medicine, 1 Illini Drive, Peoria, IL 61605, USA; 4 Department of Basic Sciences, New York University, College of Dentistry, 345 East 24th Street, New York, NY 10010, USA

## Abstract

Mitochondrial permeability transition pore (mPTP) opening allows free movement of ions and small molecules leading to mitochondrial membrane depolarization and ATP depletion that triggers cell death. A multi-protein complex of the mitochondrial ATP synthase has an essential role in mPTP. However, the molecular identity of the central 'pore' part of mPTP complex is not known. A highly purified fraction of mammalian mitochondria containing C-subunit of ATPase (C-subunit), calcium, inorganic polyphosphate (polyP) and polyhydroxybutyrate (PHB) forms ion channels with properties that resemble the native mPTP. We demonstrate here that amount of this channel-forming complex dramatically increases in intact mitochondria during mPTP activation. This increase is inhibited by both Cyclosporine A, an inhibitor of mPTP and Ruthenium Red, an inhibitor of the Mitochondrial Calcium Uniporter. Similar increases in the amount of complex formation occurs in areas of mouse brain damaged by ischemia-reperfusion injury. These findings suggest that calcium-induced mPTP is associated with *de novo* assembly of a channel comprising C-subunit, polyP and PHB.

## Introduction

The mitochondrial permeability transition pore (mPTP) is a large, weakly selective channel found in the mitochondrial inner membrane.^1^ Opening of the mPTP channel leads to a dramatic increase in the inner membrane permeability, dissipation of the mitochondrial membrane potential and disruption of ATP production that, if not reversed, can trigger cell death. mPTP activation is thought to have a major role in ischemic cell death associated with stroke and myocardial infarction. This hypothesis is supported by the fact that administration of cyclosporine A, an mPTP inhibitor, reduces infarct volume following ischemia-reperfusion injury in the heart.^2^ Although these findings suggest that the mPTP is a viable and important drug target, therapeutic efforts in this field have been hampered by a poor understanding of the molecular organization of this channel.

Compelling evidence from several laboratories suggest that the mPTP is associated with, or is an integral part, of a multi-protein complex formed by ATP synthase.^3–6^ More specifically, the C-subunit of ATP synthase, which can form channels in model membranes,^6^ appears to be essential for mPTP opening.^5^ However, the C-subunit alone cannot replicate mPTP activity suggesting that other molecules participate in this complex. The precise nature and molecular organization of the channel part of mPTP remain controversial.^7–11^

We have developed a protocol for the isolation and purification of the mitochondrial channel that closely resembles activity of native mPTP channels.^12^ This protocol, originally described by Reusch and Sadoff,^13^ is a modified solvent extraction for the isolation of the channel formed by inorganic polyphosphate (polyP), polyhydroxybutyrate (PHB) and calcium in bacteria. In the case of mammalian mitochondria, we have shown that in addition to polyP and PHB, this highly purified fraction also contains the C-subunit.^12^ This channel showed characteristics that resembled the native mPTP with multiple conductance states and a voltage dependence response. Our subsequent studies have confirmed that participation of polyP is essential for the calcium-induced activation of the mPTP.^14–17^

Our central goal was to test the hypothesis that mPTP activation is caused by calcium-induced formation of the complex of C-subunit, polyP and PHB. To achieve this objective, we compared the amounts of the complex purified from control mitochondria and mitochondria with induced mPTP activity. We found that the amount of C-subunit, polyP and PHB complex was increased in mitochondrial preparations with activated mPTP. On the basis of these results, we propose the novel concept that mPTP activation requires *de novo* calcium-induced assembly of the channel-forming complex composed of C-subunit, polyP, PHB and calcium.

## Results

### mPTP induction in isolated mitochondria

**Figure 1:**
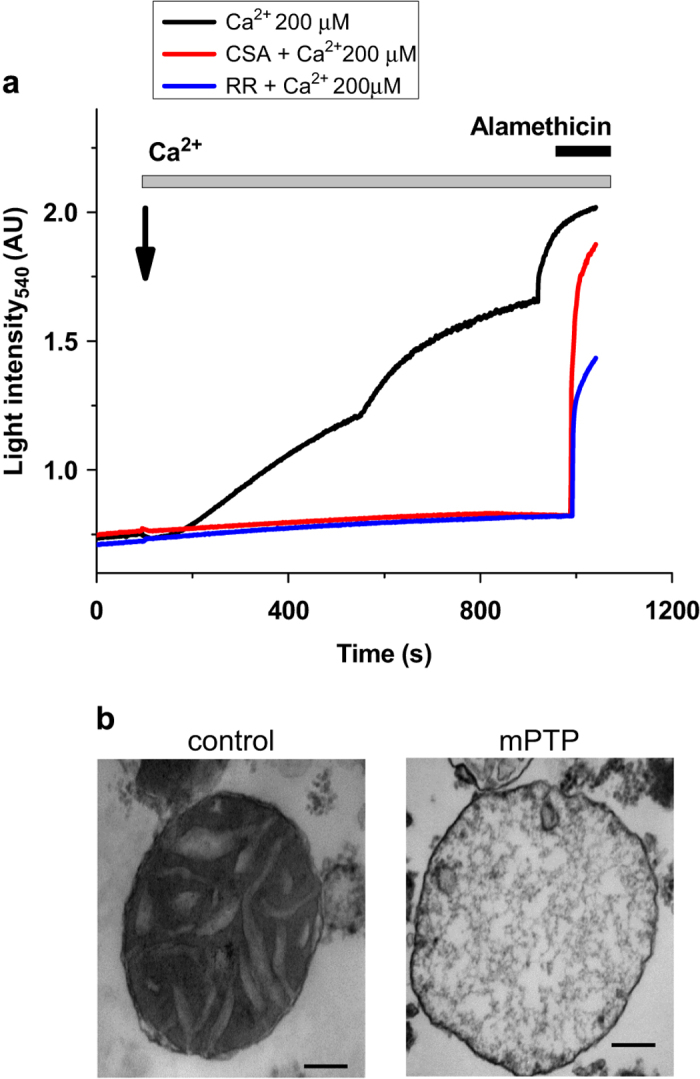
mPTP opening induced by Ca^2+^ in isolated mitochondria is blocked by CSA and Ruthenium Red. (**a**) Mitochondrial swelling induced by Ca^2+^ (detected as an increase in light transmittance) corresponds to mPTP opening (black) as this swelling is blocked by mPTP inhibitor, CSA (red) or by inhibition of the mitochondrial Ca^2+^ uptake with Ruthenium Red. Alamethicin was added at the end of the experiments to achieve maximal swelling. (**b**) Electron micrographs of condensed mitochondria before (control) and swollen mitoplasts after mPTP activation (mPTP). Scale bar is 100 nm.

Previous attempts to isolate the mPTP channel have focused on purification of the channel-forming material from isolated mitochondria that are viable and lack mPTP activity.^4,6^ Previously, we proposed the possibility that not only mPTP channel opening but also its formation might be stimulated by mitochondrial calcium uptake,^12^ which was consistent with previous works by Bernardi’s group.^18^ If this is the case, components of the channel-forming fraction should be increased in mitochondria under conditions of calcium-induced mPTP activation. To test this hypothesis, we performed the chemical analysis of the components of the channel-forming complex that demonstrates mPTP activity^12^ in control and mPTP-induced mitochondria. In these experiments, we used isolated rat liver mitochondria. Use of isolated mitochondria rather than cultured cells was advantageous as it allowed us to carefully control Ca^2+^ load and provided sufficient amount of material for biochemical assays. Before the complex extraction and purification, we induced mPTP in energized isolated mitochondria by the addition of calcium. The mPTP induction was monitored by measuring mitochondrial swelling. Figure 1 shows a representative example of such assays that were routinely performed before biochemical assays described in the following sections. This was required to establish that mitochondria have developed mPTP or that mPTP was inhibited. As can be seen from Figure 1a (black trace), the addition of 200 *μ*M of calcium to the mitochondria (1 mg protein/ml) leads to the increase in transmitted light intensity, indicative of mitochondrial swelling. We further confirmed mitochondrial swelling by electron microscopy. Figure 1b shows representative electron microscopy images of normal mitochondria with dense matrix and clearly visible cristae ('control') in comparison with the swollen mitochondria with activated mPTP (labeled as 'mPTP'). Note the typical loss of the mitochondrial outer membrane, low electron density and disrupted cristae in the mitochondria of calcium-treated samples (see Elustondo *et al*.^19^). Swelling was not detected in the presence of Cyclosporine A (CSA), an inhibitor of mPTP (Figure 1a, red trace) or Ruthenium Red, an inhibitor of mitochondrial calcium uptake (Figure 1a, blue trace) confirming the involvement of mPTP activation. Alamethicin, a pore forming ionophore, was added at the end of each experiment to confirm mitochondrial integrity. Following mitochondrial treatment and confirmation of mPTP activation, mitochondrial samples were subjected to the dehydration procedure by sequential methanol, methanol/acetone and acetone treatment followed by a chloroform extraction as detailed in the methods section.

### Analysis of the components of the channel-forming fraction purified from the calcium-treated mitochondria

As described by our previous work,^12^ the channel-forming fraction of calcium-treated mitochondria contained C-subunit of ATP synthase, polyP and PHB. This fraction showed channel activity when reconstituted in planar lipid bilayer that resembled the native mPTP with multiconductance states and voltage dependence.^12^ Here we compared the levels of all these compounds in purified control and mPTP-activated mitochondria. To make sure that equal amounts of material were used, equal volumes of the mitochondrial sample originally taken from the same tube of the isolated mitochondria were used in all the experiments presented in the figures. This allowed us to exclude any variability that might have occurred due to different amounts of mitochondria or their different functional state.

**Figure 2:**
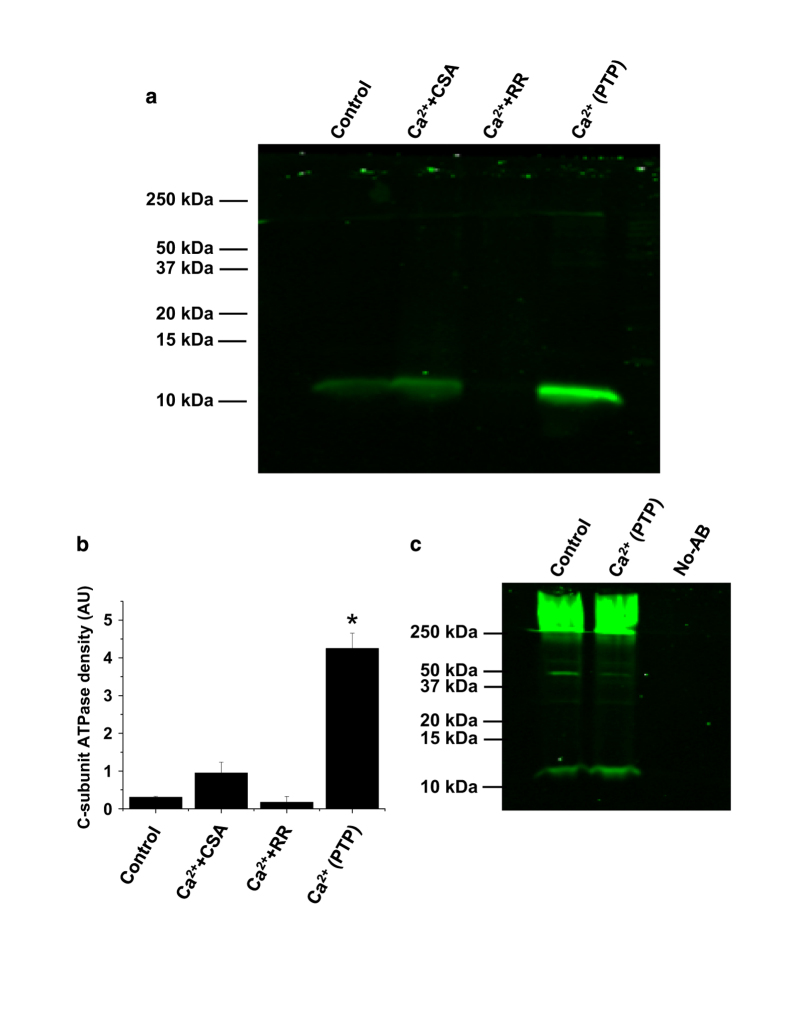
mPTP activation increases C-subunit content of the channel-forming fraction (chloroform extractable C-subunit). (**a**) Western blot using C-subunit-specific antibodies shows that mPTP activation in isolated mitochondria (+Ca^2+^) increases the amount of C-subunit associated with channel-forming 'PTP pore' fraction. Note that the amount of complex is dramatically decreased if mPTP is inhibited with 5 *μ*M Ruthenium Red (Rut Red) or 1 *μ*M CSA despite the presence of the same amounts of Ca^2+^ (200 *μ*M). The samples for analysis were purified using chloroform extraction protocol as described in Pavlov *et al*.^12^ and identical to the samples used for mPTP monitoring in Figure 1. (**b**) Densitometry analysis of western blots (as in **a**) from three independent experiments (*P<0.01 *t*-test). (**c**) Western blot of C-subunit immunoprecipitated from the total mitochondrial lysate using C-subunit antibodies shows similar total amounts of C-subunit in different samples. Note high molecular bands presumably formed by oligomers of the C-subunit.

Quantification of C-subunit in the channel-forming fraction of the extract was analyzed using immunoblotting assays with C-subunit antibodies. As can be seen from Figures 2a and b, the extracts from mitochondria with activated mPTP contained significantly higher amounts of C-subunit compared with control mitochondria (*n*=3, *P*<0.01). Importantly, when mitochondria were treated with the same amount of calcium in the presence of the mPTP inhibitor Cyclosporine A, C-subunit levels were not increased compared with the C-subunit levels detected in control and Cyclosporine A-treated mitochondria. Similarly, C-subunit levels were not increased when calcium treatment was done in the presence of Ruthenium Red – an inhibitor of mitochondrial calcium uptake. These results support the idea that the transfer of C-subunit into the chloroform extract is directly related to mitochondrial calcium uptake and activation of the mPTP. Taking into account that with the exception of C-subunit this extraction protocol leads to the sample with non-detectable levels of protein,^12^ it was not possible to use endogenous protein as a loading control. To make sure the samples were comparable, the total amount of extract, which in each experiment is originated from the same amount of mitochondria, was resuspended in equal volumes and run on the same gel. To confirm that the increase in the detection of C-subunit was not a consequence of a change in the amount of total protein occurrence as a consequence of the Ca^2+^ treatment, we studied the amount of C-subunit directly immunoprecipitated from the whole mitochondrial lysates exposed to high or no Ca^2+^. As can be seen from the immunoblot results, the total amount of C-subunit did not change after calcium treatment (Figure 2c). These control experiments demonstrate that there is no change in the total C-subunit levels present in the sample regardless of the treatment condition. We were also concerned that the recovery of higher amounts of C-subunit could be a consequence of a difference in the membrane integrity caused by the mitochondrial swelling of the preparation so we included a control where we lyzed the mitochondria with a buffer containing detergent (500 mM NaCl, 50 mM NaH_2_PO_4_, 20 mM Hepes and 10% glycerol, pH 7.4). We did not detect an increase in C-subunit in the chloroform extract from this preparation (not shown). The increase in the relative amount of C-subunit detected in induced mPTP fractions, but not in control fractions, supports a role for the C-subunit in the mPTP channel-forming fraction of mitochondria.

**Figure 3:**
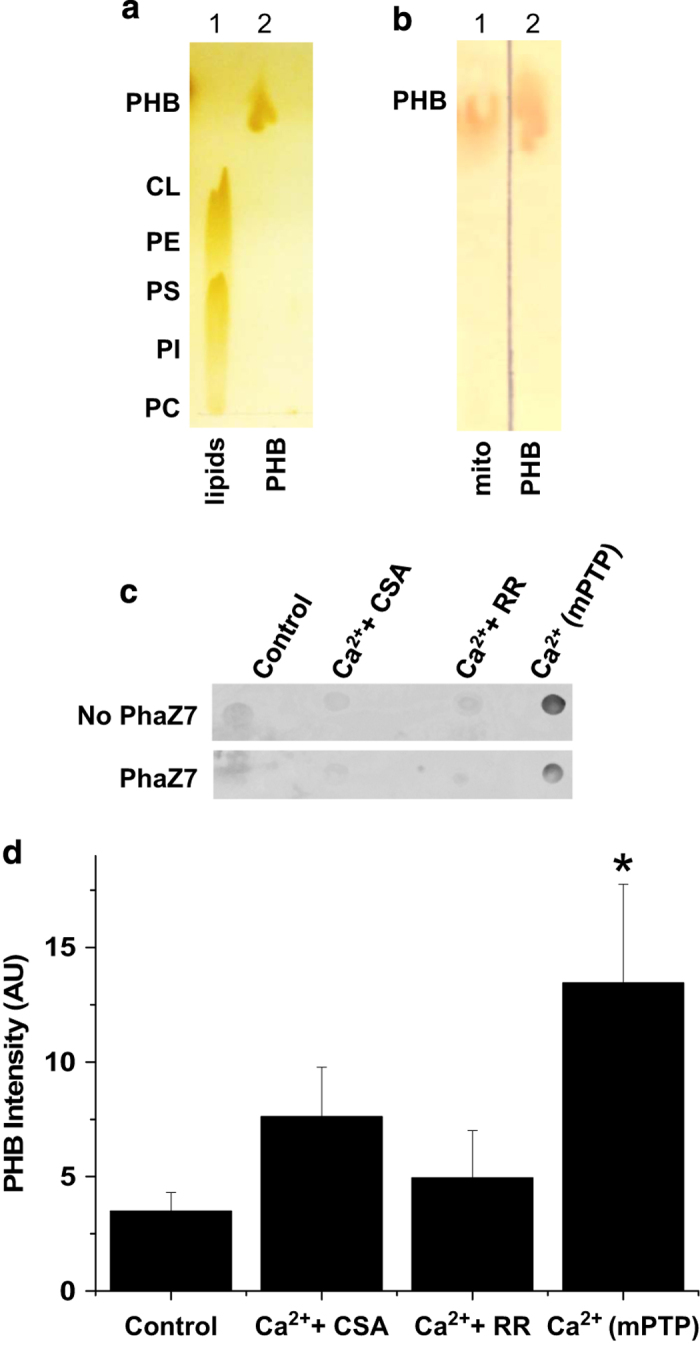
PHB is present in channel-forming fraction and is increased upon activation of mPTP. (**a**) Thin-layer chromatography (TLC) showing a mix of phospholipid standards (lane 1, CL: cardiolipin; PE phosphatidylethanoamine; PI, phosphatidylinositol; PS, phosphatidylserine) and synthetic PHB standard (lane 2). (**b**) TLC showing mitochondrial extract (lane 1) and a PHB standard (lane 2). Both TLCs were stained with Iodine. (**c**) PHB detection using dot blot immunostaining of the extracts using PHB antibodies. Note that the signal was reduced by PHB-depolymerase (PhaZ7) treatment. PHB was present in higher amounts in calcium-treated mitochondria -Ca^2+^(mPTP)- compared with control or mitochondria pretreated with Cyclosporin A (+CSA) or Ruthenium Red (+RR). (**d**) Densitometry analysis of dot blots from four independent experiments showing a significant increase in the recovery of PHB in the extract from mitochondria treated with calcium when compared with control, RR- or CSA-treated mitochondria (*n*=4, *P<0.05, *t*-test).

**Figure 4:**
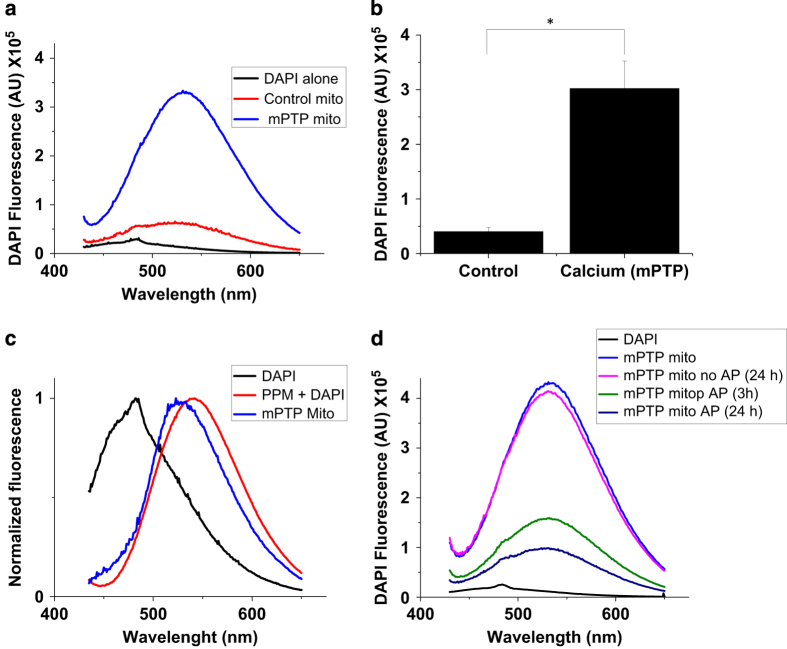
mPTP activation increases polyP levels in the channel-forming fraction. (**a**) Emission spectra of DAPI in the presence of the extracts purified from mitochondria treated with 10 mM Ca^2+^ to induce mPTP (blue) and control (red). The black line shows the emission spectrum of DAPI alone. (**b**) Combined data of the values of peak DAPI fluorescence from three independent experiments. Note the significant increase in the amount of polyP in treated calcium samples (blue) when compared with control (red; *t*-test, *P<0.002). (**c**) Comparison of the emission spectra of DAPI alone (black), synthetic polyP (PPM, 60 phosphates, red) and purified fraction (blue); (**d**) Alkaline phosphatase (AP) treatment shows a decrease in the DAPI fluorescence confirming the presence of polyP.

Next, we analyzed the amount of PHB in the chloroform extracts. In contrast to polyP, PHB is not soluble in water and can only be dissolved in nonpolar organic solvents. First, we used thin-layer chromatography (TLC) to separate the nonpolar content of the chloroform extract. Figure 3a shows the relative distribution of synthetic phospholipids and synthetic PHB standard on the TLC plate. Due to its higher hydrophobicity, PHB migrates significantly faster compared with phospholipids. As can be seen from Figure 3b, the chloroform extract contained only the material that migrated at the same rate as the PHB standard. In both Figures 3a and b, this material was visualized by nonspecific iodine staining. On the basis of the TLC, the retention factors (Rf) of the hydrophobic material in the channel-forming fraction (Rf 0.6–0.9) were similar to the PHB standard (Rf 0.6–0.8). To confirm the presence of PHB, we blotted the sample onto a PVDF membrane and visualized this molecule using PHB-specific antibodies. Figures 3c and d show that the channel-forming fraction from mPTP-activated mitochondria contained significantly higher amounts of PHB compared with samples isolated from control mitochondria or from mitochondria in which the mPTP was inhibited with either CSA or ruthenium red treatment. Signal intensity was decreased by treatment of the sample with a specific PHB-hydrolyzing enzyme (PHB depolymerase, PhaZ7^(ref.20)^) further confirming that the detected signal reflects the presence of PHB (Figure 3c). Finally, we measured the amounts of polyP in the chloroform extracts of control and calcium-treated mitochondria. To detect polyP, the extract was transferred from a chloroform soluble ('complex' form) phase into a water soluble ('free' form) phase. This was achieved by the incubation of the chloroform phase with water. This procedure allows dissociation of the PHB/polyP complex, transfer of the polyP into the polar water phase and retention of PHB in the chloroform phase. PolyP was detected in the water phase with a DAPI-based fluorescence assay. When polyP binds to DAPI, it induces a shift in the excitation and emission peaks characteristic for polyP.^21^ As can be seen from Figures 4a and b, the amount of polyP was significantly higher in the samples from calcium-treated mitochondria compared with control mitochondria. The fluorescent spectrum of the sample was similar to the spectrum for synthetic polyP (Figure 4c). On the basis of the calibration curve performed using polyP standards, we estimate that the amount of polyP extracted from 1 g of liver is approximately 1 *μ*g. The fluorescent signal was eliminated from the sample by treatment with alkaline phosphatase to induce polyP hydrolysis (Figure 4d) thus confirming that it reflects the amount of polyP present in the sample. However, the increase in polyP was only observed when the process of dehydration was done in the presence of high amounts of calcium (10 mM). We hypothesize that polyP is required in the process of pore formation but is easily dissociated from the complex as soon as the local concentration of calcium decreases following mPTP activation and its efflux from the matrix. PolyP is a highly charged polyanion with excellent water solubility making extraction with chloroform only possible if this polymer is complexed with hydrophobic molecules. We have previously shown that the ability of polyP to be extracted with chloroform from mPTP-activated mitochondria is determined by the presence of PHB.^12^ Altogether, these findings indicate that the induction of mPTP activity in mitochondria is accompanied by increased levels of PHB, polyP and C-subunit known to have channel-forming activities.

### Direct extraction of C-subunit by chloroform from Hypoxic-Ischemic-injured brain

The use of isolated mitochondria allowed us to establish the direct link between Ca^2+^ mPTP and formation of the PHB, polyP and C-subunit complex. The activation of the mPTP is thought to have a pivotal role in the pathophysiology of ischemia-reperfusion injuries. For this reason, we utilized an ischemia-reperfusion model of stroke in mice to investigate the contributions of polyp, PHB and c-subunit *in vivo*. The activation of mPTP is considered to have a central role in ischemic cell death responsible for brain damage after a stroke (reviewed in Bernardi *et al*.^22^). We investigated whether increased mPTP channel formation, observed at the level of isolated mitochondria, is also induced by HI brain damage in mice. Before the use of this animal model, we tested the feasibility of the extraction method by analyzing the channel-forming fraction purified directly from the whole liver without prior mitochondrial isolation. The amount of C-subunit in chloroform extracts were examined in whole livers homogenized in the presence and absence of EGTA. In the absence of EGTA during homogenization, mitochondria are exposed to high levels of calcium that induce opening of mPTP. Analysis of the extracts by dot blot using C-subunit antibodies (Supplementary Figure 1) showed that in the presence of high calcium, C-subunit levels in extracts from whole liver were significantly higher compared with those observed in control extracts isolated from tissues homogenized in the presence of EGTA. Consistently higher amounts of PHB were recovered in the extracts from calcium-exposed livers. The presence of PHB was confirmed by the decrease in signal when the samples were treated with a specific PHB depolymerase, PhaZ7 (Supplementary Figure 1B).

**Figure 5:**
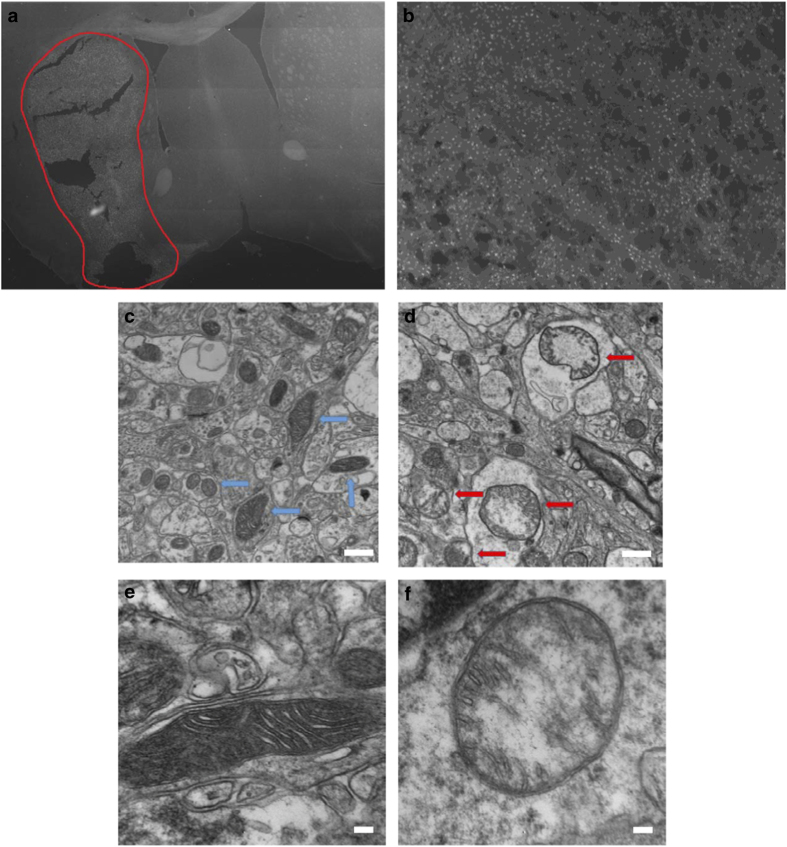
Characterization of the cell and mitochondrial damage following ischemia. (**a**) Representative 10×10 tile stained with Fluoro-Jade to identify degenerating neurons. The picture is representative of the damage observed in the striatum of animals following HI-induced brain injury. The damaged area is outlined by the red line. (**b**) Image of an individual tile at ×10 magnification representative of the damage observed in the striatum. (**c**) EM image of control brain tissue with fully intact mitochondria marked with blue arrows. (**d**) EM image of damaged area showing damaged mitochondria with loss of normal cristae structure, marked by red arrows. (**e** and **f**) EM images of control and damaged mitochondria at increased magnification. Scale bars are 500 nm for **c** and **d**; 100 nm for **e** and **f**.

**Figure 6:**
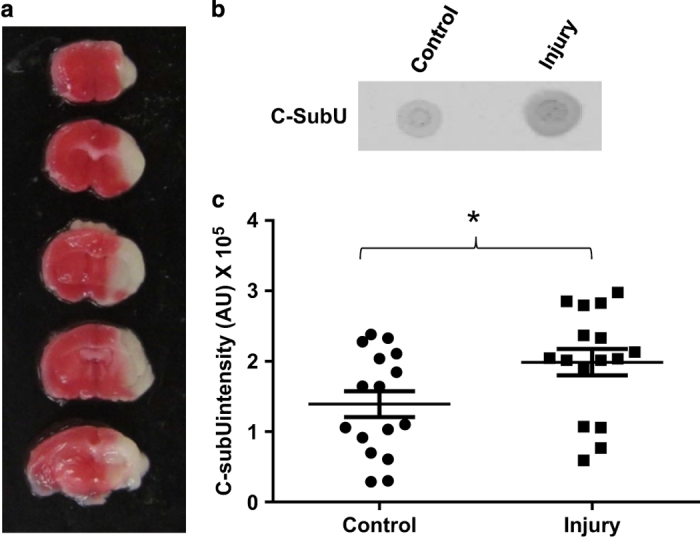
HI brain injury is associated with increased C-subunit levels in the channel-forming fraction. (**a**) Image of a whole brain stained with TTC (2,3,5-triphenyltetrazolium chloride) from a representative mouse subjected to HI brain injury. Viable tissue is stained red while the infarct is unstained or white. (**b**) Dot blot for C-subunit prepared using C-subunit antibodies. The extracts from the injured side of the brain showed higher amounts of C-subunit compared with the non-injured side. (**c**) Densitometry analysis of the C-subunit dot blots of chloroform extracts from brain uninjured (control) and damaged (injury) hemispheres showing increased C-subunit levels in the damaged hemisphere (*P<0.05, Mann–Whitney nonparametric test, *n*=16 animals).

On the basis of these results, we hypothesized that the amount of C-subunit recovered in a water-free chloroform extract from the HI-injured hemisphere would be greater relative to that obtained from the non-injured hemisphere. To test this hypothesis, mice were subjected to unilateral common carotid artery occlusion followed by exposure to a low oxygen (8% O_2,_ 50 min) environment to induce HI brain injury. Fluoro-Jade (FJ) staining, which positively labels degenerating neurons, was performed to quantify the level of neuronal damage 24 h following HI. A representative tiled image at the level of the midstraitum is shown to demonstrate the size of the (Figure 5a). Figure 5b shows FJ positive staining in the damaged striatum. Mitochondrial injury was also assessed by direct visualization of mitochondria by electron microscopy. EM images of control hemisphere (Figures 5c and e) display mitochondria with well-formed cristae and double-membrane structures. In contrast, the mitochondria from the infarcted hemisphere display a loss of cristae and membrane integrity (Figures 5d and f). TTC staining indicated that the infarct produced by HI occupied about 50–70% of the affected hemisphere (Figure 6a). The contralateral (non-injured) and ipsilateral (injured) brain hemispheres from mice subjected to unilateral HI injury were collected in methanol and extracted using the water-free chloroform extraction method. The extracts were analyzed by dot blot with anti-C-subunit and anti-PHB antibodies. Figure 6b is a representative image from an HI-injured mouse showing that relative to an extract from the uninjured (control) hemisphere, the amount of C-subunit appears to be elevated in the injured hemisphere. This finding was confirmed by the quantification of C-subunit levels in the control and injured hemispheres of 16 HI mice (Figures 6c, *P*<0.05, Mann–Whitney nonparametric test). Interestingly, there was no significant difference in the amount of PHB recovered in these samples when the injured and non-injured hemispheres were compared (Supplementary Figure 1C). This result may reflect the high abundance of PHB in brain.

## Discussion

Recent models of mPTP generally agree that ATP synthase has an essential role in formation of the mPTP.^3,8,9^ Moreover, genetic manipulations of the C-subunit of ATP synthase correlate with alterations in mPTP activity.^6^ This suggests that the C-subunit of the ATP synthase is an essential component of the mPTP channel. The C-subunit is an oligomer that forms a barrel-like structure in the lipid bilayer that could comprise the mPTP pore. Consistent with this proposal, purified C-subunit can form ion-conducting pores albeit their characteristics do not closely resemble native mPTP activity.^23^ We showed, in previous work, that PolyP is also required for mPTP opening in primary neuronal cultures and cardiac myocytes.^16,17^ Furthermore, when added to cultured cells, PHB is targeted to the mitochondria where it induces mPTP.^24^ Our demonstration that comparable elevations of C-subunit of ATPase, polyP and PHB were detected in chloroform extracts from mPTP-activated mitochondria supports an important role of these polymers in mPTP activity. By contrast, when Ca^2+^-induced mPTP activation was blocked with the Ca^2+^ uptake inhibitor Ruthenium Red, C-subunit was significantly lower in the extract. The reliance of channel formation on mitochondrial calcium uptake suggests that Ca^2+^ is exerting its effect in the mitochondrial matrix space. Inhibition of the Ca^2+^-induced mPTP activation with Cyclosporine A also reduced C-subunit levels in the chloroform extract. The *in vivo* relevance of these findings is supported by the elevation of C-subunit levels in chloroform extracts from the damaged hemisphere after an experimental stroke. In this animal surgical model, the stroke was induced by restricting the blood circulation to one hemisphere of the brain, followed by 50 min of hypoxia. This protocol induces the opening of mPTP.^25^ On the basis of these results, we propose that formation of the mPTP requires a calcium-induced assembly of the complex composed of C-subunit, polyP and PHB. These findings suggest that induction of the mPTP is linked to a change in C-subunit association with these polymers rather than a change in the total amount of the C-subunit or in its conformation. This interpretation likely explains why the C-subunit is not found in the purified fraction of the control mitochondria. Indeed, when C-subunit is not complexed with PHB and polyP, this protein likely dissolves in the acetone phase during the dehydration step and is therefore not present in the pellet used for channel extraction by chloroform. In contrast, when the C-subunit associates with polyP and PHB, this protein is rendered soluble in the chloroform phase.

The essential requirement of interaction between three components (C-subunit, polyP and PHB) to form the functional mPTP provides a mechanism to resolve several controversial points:

1. C-subunit can form large pores of eight C-subunits in mammalian cells.^26^ However, the C-subunit is a highly hydrophobic protein that is not expected to be able to form water-filled pores. This suggests that, on its own, the C-subunit probably cannot assemble into the functional mPTP. Furthermore, unlike native mPTP, the C-subunit alone is not expected to have significant voltage dependence owing to the lack of charged groups necessary to gate ion conductance. The ability of polyP to generate channels with significant voltage dependence and selectivity^27–29^ suggests that this charged polymer may serve as the hydrophilic lining of the mPTP pore largely responsible for its key electrophysiological properties. Furthermore, our previous studies have shown that the same channel demonstrates a transition from a large pore resembling C-subunit channel into a small channel resembling activity of the synthetic polyP/PHB channel in a voltage-dependent manner.^12^ The molecular function of channels that involve PHB, polyP and calcium have been described in details and confirmed experimentally by Reusch and Seebach.^28,30^ We hypothesize that similar principles govern the function of the mitochondrial mPTP channel, but in this case, the polyP/Ca^2+^/PHB channel is likely formed association with the C-subunit ring.
2. It is known that activation of mPTP is caused by an excessive accumulation of calcium. Despite this calcium accumulation, mPTP can be activated while concentrations of free calcium remain constant due to the formation of calcium phosphate complexes.^31^ A recent study indicates that polyP might have an important role in the regulation of this process.^32^ Thus, at the level of intact mitochondria, mPTP is not a channel directly activated by a simple increase in bioavailable calcium but rather by calcium phosphate species. Taking into account that polyP is a 'condensed' form of phosphates and that there is an increase in the formation of calcium phosphate complexes before mPTP opening, it is tempting to speculate that calcium polyP, rather than calcium phosphate interactions, are the direct cause of mPTP formation and opening. More detailed discussion of this model in comparison with other current views can be found in Solesio *et al*.^33^
3. Finally, patch-clamp studies indicate that very few mPTP channels are present in the mitochondria.^34,35^ However, mitochondria contain high amounts of C-subunit oligomers, which is inconsistent with the relatively rare detection of mPTP. As can be seen from Figure 2, only a small amount of the C-subunit is associated with polyP in the fraction capable of forming mPTP-like channels. We hypothesize that only a small amount of C-subunit participates in complex formation with polyP and PHB. This proposal provides an explanation for the discrepancy between the large amount of C-subunit and the small amount of mPTP channels per mitochondrion.

In conclusion, our results suggest that the assembly of a complex composed of the C-subunit, polyP and PHB is required for mPTP opening which is induced by calcium in the case of isolated mitochondria or by HI brain damage, presumably also by a calcium overload mechanism. We propose that activation of the mPTP is linked to the formation of the *de novo* complex, which is not normally present in functional mitochondria. Elucidation of the components of the mPTP provides a model that will assist the development of therapeutics for the prevention of ischemic brain or heart injury.

## Materials and methods

### Animals

Sprague Dawley rats and C57bl/6 mice were purchased from Charles River (Montreal, Quebec, Canada) and housed in a climate-controlled environment with appropriate light–dark cycles. All experimental animals were allowed to eat standard chow and drink water *ad libitum*. All the procedures were approved by the Animal Care Committee of Dalhousie University in accordance with standards of the Canadian Council on Animal Care. Isolated mitochondria were obtained from rats (6–10 weeks of age) killed with isoflurane/CO_2_.

### Antibodies

The following antibodies were used: anti APTase Subunit C (ab181243, Abcam, Cambridge, MA, USA); anti-PHB (gift from Dr Reusch, Michigan State University); Alexa Fluor_ 680 goat antimouse IgG (catalog number A21057, Invitrogen, Carlsbad, CA, USA); Alexa Fluor_ 750 goat anti-rabbit IgG (catalog number A21039, Invitrogen) and anti-ATPase immunocapture antibody (ab1099867, Abcam, Cambridge, UK).

### Isolation of mitochondria

Mitochondria were isolated from the liver of Sprague Dawley rats by differential centrifugation as previously described.^19^ Briefly, liver from rats was homogenized using Teflon-glass homogenizer and resuspended in 50 ml of isolation buffer (300 mM Sucrose, 5 mM Hepes-KOH, 5 mg/ml BSA, 0.2 mM KH_2_PO_4_, 1 mM EDTA, pH 7.4). An initial centrifugation at 600 g for 15 min was followed by centrifugation of the supernatant at 6500×*g* for 20 min at 4 °C. The resulting pellet was washed with buffer without EDTA and resuspended in 1 ml of washing buffer (300 mM sucrose, 5 mM Tris-ClH, 7.4).

### mPTP complex induction and extraction from mitochondria

Isolated mitochondria were resuspended in recording buffer (70 mM sucrose, 230 mM mannitol, 5 mM Hepes-KOH, 1 mM KH_2_PO_4_, supplemented with 0.8 *μ*M Rotenone, 5 mM Succinate, pH 7.4). The concentration of calcium needed to be added to the mitochondrial suspension for mPTP activation was determined, after each isolation, by monitoring swelling and was generally in the range of 100–200 *μ*M. Chloroform extraction of the complex was performed as previously described.^12^ Briefly, isolated mitochondria were resuspended in methanol, incubated for 10 min on ice, and then spun down at 4700 ×*g* for 20 min (twice). The procedure was repeated with methanol/acetone, 1:1 (twice), and then with acetone (twice). Traces of acetone were removed from the final pellet with a stream of dry nitrogen. Typically, chloroform extraction was done by incubating the dried pellet in 5 ml chloroform at 4 °C for 48–72 h in Teflon tubes (Nalgene, Rochester, NY, USA). All solvents used during the process of dehydration and extraction were ice cold, and were pretreated with molecular sieve beads, 8–12 mesh (No. 208604, Sigma-Aldrich, St Louis, MO, USA) to eliminate all traces of water. All the procedures were performed on ice. Before the extracted fraction was resuspended in sample buffer, the chloroform suspension was filtered with a 0.2 mm PTFE filter (Acrodisc Syringe Filter, PALL Life Sciences, East Hills, NY, USA) using a Teflon syringe. The filtered extract was dried under a nitrogen stream and resuspended in the appropriate buffer for the experiment.

### Protein assay

Isolated mitochondria were resuspended in 100 *μ*l of lysis buffer consisting of (in mM): 50 Tris, 150 NaCl, 50 Na_2_HPO_4_, 1 Na_3_VO_4_, 1 NaF and 0.1% Nonidet P-40, and 0.25% sodium deoxycholate. The lysis buffer also contained 1 *μ*l of a protease inhibitor mixture (Sigma-Aldrich, catalog number P8340). The samples were homogenized and incubated at 4 °C with constant agitation for 30 min, then centrifuged at 12 000×*g* for 15 min. Protein concentration was determined in the supernatant of each sample with a modified Lowry method (Bio-Rad, Hercules, CA, USA; DC Protein Assay, catalog number 500-0116).

### Thin-layer chromatography

TLC was performed on silica gel G (Analtech, Newark, DE, USA). Mitochondrial extracts were resuspended in 50 *μ*l chloroform and 10 *μ*l aliquots were applied to the TLC plates. The plates were analyzed using chloroform:methanol:water (90:10:0.5) as the solvent system. The staining was performed with iodine vapors.

### Immunoblot analysis

Following protein quantification (DC Protein Assay, Bio-Rad, catalog number 500-0116), 200 *μ*g of protein was boiled with loading buffer with 5% *β*-Mercaptoethanol and separated with12% SDS-PAGE gels and transferred to polyvinylidene fluoride membranes (catalog number IPFL00010 Immobilon, FL, Millipore, Billerica, MA, USA). In the case of the extracts, the samples were resuspended in 25 *μ*l of 100 mM Tris buffer, 0.05% SDS, pH 7.4 The membranes were stained with Ponceau red to assess loading; after they were rinsed with Tris-buffered saline/Tween 20 (TBST; 20 mM Tris base, 137 mM NaCl and 0.1% Tween 20, pH 7.6), the blots were blocked in blocking buffer (Oddissey catalog number 927–40000) for 1 h. The blots were incubated with antibodies for ATP synthase subunit C (catalog number ab47010, Abcam), overnight at 4 °C with constant agitation. Following three washes with PBST, the immunoblots were incubated with either Alexa Fluor 680 goat antimouse IgG (catalog number A21057, Invitrogen) or Alexa Fluor 750 goat anti-rabbit IgG (catalog number A21039, Invitrogen) depending on the primary antibody used. The bands were detected with Odyssey version 3.0, and multiple exposures were generated to ensure the linearity of the fluorescent signal.

Immunoprecipitation was performed with Pierce (Waltham, MA, USA) IgA magnetic beads (catalog number 88846) according to a standard protocol.^36^ Briefly, 500 *μ*g of proteins from the whole mitochondrial lysate were incubated overnight with the anti-ATP synthase IgG conjugated to protein A magnetic beads (catalog number 88846, Pierce, Thermo Scientific. The samples were then boiled in SDS-loading buffer and equal amounts of sample were loaded in each lane including a no-antibody control. Immunoblot analysis was done with the anti-subunit-C antibody.

### Transmission electron microscopy

Two hours following HI, the mice were humanely killed with an overdose of pentobarbital (150 mg/kg). The brains were perfused with PBS followed by fixation in 2.5% glutaraldehyde in PBS (pH 7.4) and processed by the EM Core Facility (Dalhousie University). In short, the samples were rinsed 3× in 0.1 M sodium cacodylate buffer and fixed in 1% osmium tetroxide for 2 h, then dehydrated and embedded in epon araldite resin. Hundred nanometer sections were cut with an ultramicrotome and placed on 300-mesh copper grids. The sections were stained with 2% aqueous uranyl acetate, rinsed and treated with lead citrate, rinsed again and air dried. The images were captured with a Jeol Jem 1230 transmission electron microscope at 80 kV attached to a Hamamatsu (Middlesex, NJ, USA) ORCA-HR digital camera.

### FJ staining

The mice were injected with a lethal overdose of sodium pentobarbital (150 mg/kg, i.p.) and perfused transcardially with PBS (5 ml) followed by 0.1 M phosphate buffer containing 4% paraformaldehyde (pH=6.5; 5 ml) to fix the brain. The brain was removed and post-fixed in 0.1 M phosphate buffer containing 4% paraformaldehyde for 24 h and cryoprotected for 48 h in 0.1 M phosphate buffer containing 30% sucrose. The brain was then frozen and 30 *μ*m coronal sections were cut through the forebrain. The sections were stained with FJ (AG325-30MG; EMD Millipore (Darmstadt, Germany), Canada) according to the manufacturer’s recommendations. The slides were then cover-slipped with Fluorescence-preserving VECTASHIELD Mounting medium (Vector, Burlingame, CA, USA; H-1,000) and observed under a Zeiss (Thornwood, NY, USA) fluorescence microscope equipped with a computer-assisted image analysis system. The picture shown is representative of the damage observed in the striatum.

### Mouse stroke model

C57bl/6 mice were used for the study. Ethical approval for the study was obtained from the Institutional Animal Care Committee (Dalhousie University). Hypoxic-ischemic (HI) brain injury was produced using a method adapted from Levine.^37^ The mice were anesthetized using isoflurane (3% with medical oxygen at a flow rate of 3 l/min). The ventral portion of the neck was shaved and cleaned with soluprep and betadiene (SoluMed Inc; Laval, QC, Canada and Purdue Frederick Inc; Pickering, ON, Canada, respectively). Anesthesia was maintained with 2% isoflurane with an oxygen flow rate of 1.5 l/min. A 1 cm incision was made with a scalpel to expose the sternohyoid and sternomastoid muscles. The left carotid artery was isolated and removed from the vagus nerve by blunt dissection. The carotid was then permanently occluded using a high-temperature electrocautery pen (Bovie Instruments; St. Perersberg, FL, USA). Incomplete occlusions and/or animals that displayed blood loss were immediately killed and excluded from the study. Following a 2–3 h recovery period, the animals were placed in a low-oxygen chamber (8% oxygen balanced with nitrogen, flow rate 4 l/min). Following 50 min in the low-oxygen chamber, the mice were removed and individually housed to recover. Following a 4 h recovery, the mice were selected either for TTC (2,3,5-triphenyltetrazolium chloride, St. Louis, MO, USA) or water-free chloroform extraction. In both cases, the mice were humanely killed by intraperitoneal injection of an overdose of sodium pentobarbital overdose (100 mg/kg) and perfused intracardially with PBS. The mice were then decapitated and their brains rapidly removed from the cranial vault. For TTC analysis, the brains were then placed in brain blocks and stored at −20° C for 5 min. Next, the brains were sliced into 1 mm coronal sections and placed in a 2% TTC solution at 37 °C for 15 min. The brain slices were then transferred to 4% formaldehyde for 20 min. The slices were finally stored in 1% formaldehyde in PBS until photographic analysis. The brains used for extraction were harvested in the same manner as those for TTC staining. Once perfused and removed from the skull, the hindbrain and rostral forebrain were discarded. The brain was then cut along medial longitudinal fissure, dividing the brain into the affected and unaffected hemispheres. The brain sections were then immediately placed into methanol until further processing. The brain extraction was performed using the same protocol described for mitochondrial isolation.

## Abbreviations

C-subunit: C-subunit of the mitochondrial ATP synthase
CSA: Cyclosporine A
mPTP: Mitochondrial Permeability Transition Pore
polyP: inorganic Polyphosphate
PHB: polyhydroxybutyrate
TCL: thin layer chromatography.
