# Supplementary material for: Mitochondrial permeability transition pore induction is linked to formation of the complex of ATPase C-subunit, polyhydroxybutyrate and inorganic polyphosphate
Source: Cell Death Discov. 2016 Dec 5;2:16070–. doi: 10.1038/cddiscovery.2016.70 (PMC5137186; doi:10.1038/cddiscovery.2016.70)
Supplement: Supplementary Information [file cddiscovery201670-s1.pdf]

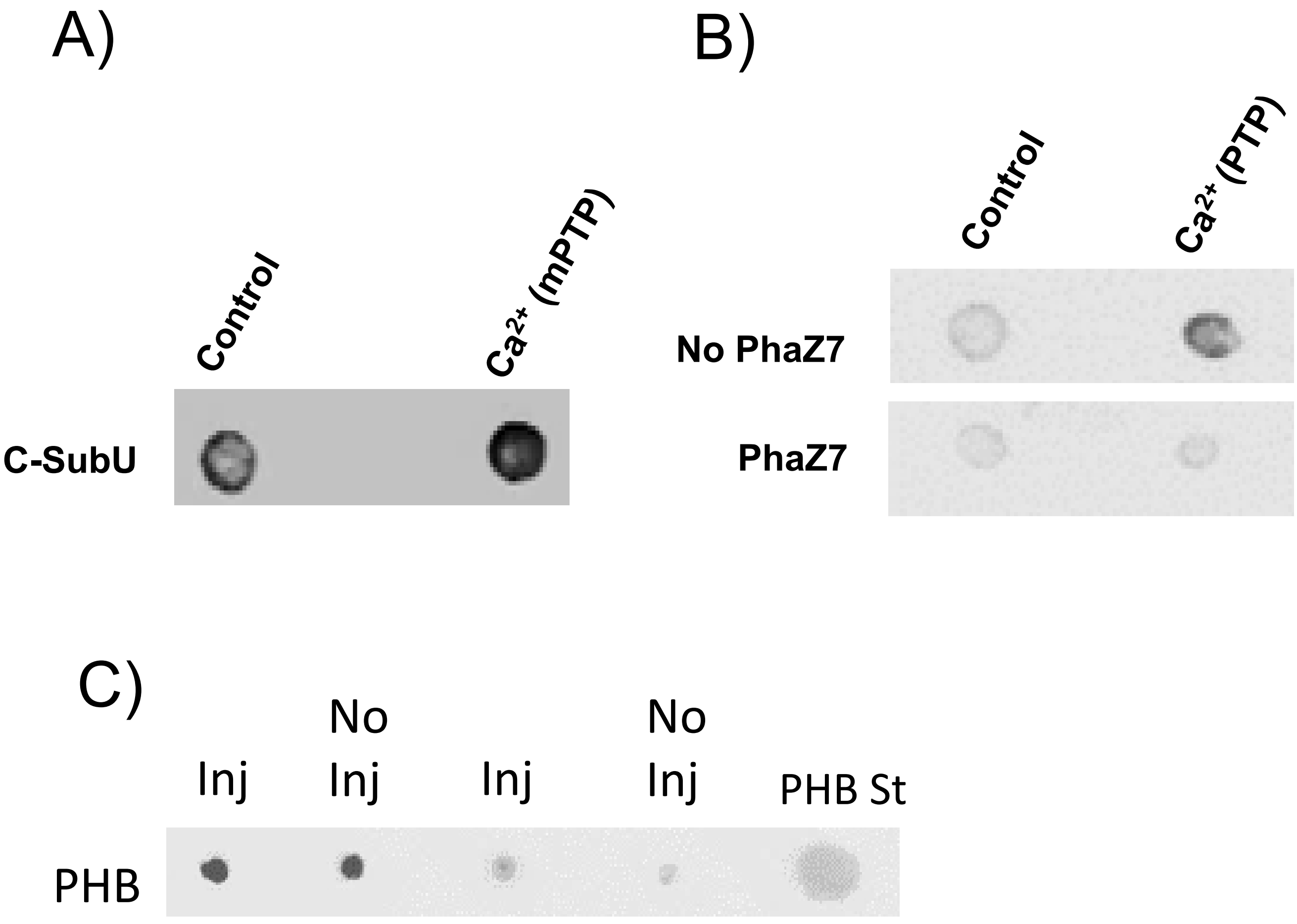

Supplementary figure 1. A) When material is extracted from total liver homogenate, the amount of C- subunit obtained in the extract is higher when homogenization is done in the presence of calcium. B) Higher amounts of PHB are recovered from extracts from rat livers homogenized in the presence of Ca^{2+} compared to livers homogenized without calcium (Control). A specific PHB depolymerase (PhaZ7) reduced the detection of PHB in both samples C)) Comparable amounts of PHB are extracted from brain hemispheres that suffered an injury from the stroke (Inj) compared to the other hemisphere (No Inj); PHB (0.2 mg) was blotted as a control.
